# Gastric emptying of a glucose drink is predictive of the glycaemic response to oral glucose and mixed meals, but unrelated to antecedent glycaemic control, in type 2 diabetes

**DOI:** 10.1038/s41387-024-00264-8

**Published:** 2024-04-08

**Authors:** Chunjie Xiang, Yixuan Sun, Yong Luo, Cong Xie, Weikun Huang, Zilin Sun, Karen L. Jones, Michael Horowitz, Christopher K. Rayner, Jianhua Ma, Tongzhi Wu

**Affiliations:** 1 School of Medicine, Southeast University, Nanjing, 210009 China; 2 Adelaide Medical School and Centre of Research Excellence (CRE) in Translating Nutritional Science to Good Health, The University of Adelaide, Adelaide, 5000 Australia; 3 Department of Endocrinology, Nanjing First Hospital, Nanjing Medical University, Nanjing, 210029 China

**Keywords:** Type 2 diabetes, Nutrition

## Abstract

**Background:**

Gastric emptying (GE), with wide inter-individual but lesser intra-individual variations, is a major determinant of postprandial glycaemia in health and type 2 diabetes (T2D). However, it is uncertain whether GE of a carbohydrate-containing liquid meal is predictive of the glycaemic response to physiological meals, and whether antecedent hyperglycaemia influences GE in T2D. We evaluated the relationships of (i) the glycaemic response to both a glucose drink and mixed meals with GE of a 75 g glucose drink, and (ii) GE of a glucose drink with antecedent glycaemic control, in T2D.

**Methods:**

Fifty-five treatment-naive Chinese adults with newly diagnosed T2D consumed standardised meals at breakfast, lunch and dinner with continuous interstitial glucose monitoring. On the subsequent day, a 75 g glucose drink containing 150 mg ^13^C-acetate was ingested to assess GE (breath test) and plasma glucose response. Serum fructosamine and HbA1c were also measured.

**Results:**

Plasma glucose incremental area under the curve (iAUC) within 2 hours after oral glucose was related inversely to the gastric half-emptying time (T50) (*r* = −0.34, *P* = 0.012). The iAUCs for interstitial glucose within 2 hours after breakfast (*r* = −0.34, *P* = 0.012) and dinner (*r* = −0.28, *P* = 0.040) were also related inversely to the T50 of oral glucose. The latter, however, was unrelated to antecedent fasting plasma glucose, 24-hour mean interstitial glucose, serum fructosamine, or HbA1c.

**Conclusions:**

In newly diagnosed, treatment-naive, Chinese with T2D, GE of a 75 g glucose drink predicts the glycaemic response to both a glucose drink and mixed meals, but is not influenced by spontaneous short-, medium- or longer-term elevation in glycaemia.

## Introduction

Gastric emptying (GE) is a key determinant of the glycaemic response to carbohydrate in both health and diabetes [1–4], such that even modest differences in the rate of GE may have a major impact on the postprandial blood glucose profile, particularly in type 2 diabetes (T2D) where, as a result of impaired glucose tolerance, the effect of GE is more sustained [5, 6]. In T2D, dietary and pharmacological interventions that slow GE (e.g., nutrient ‘preloads’ consumed before the main meal [7–9] and glucagon-like peptide-1 receptor agonists (GLP-1RAs) [10]) attenuate the glycaemic response to carbohydrate-containing meal, while the acceleration of GE (e.g., by intravenous erythromycin) increases the postprandial glycaemic excursion [11]. Moreover, the rate of GE in T2D is predictive of postprandial glucose-lowering in response to specific therapies. For example, the reduction in postprandial glycaemia induced by GLP-1RAs [12] and dipeptidyl peptidase-4 (DPP-4) inhibitors [13, 14] is greater in those T2D patients in whom GE is relatively more rapid at baseline. Accordingly, assessment of GE is of major relevance to the management of T2D.

In health, GE of nutrients usually occurs at a relatively constant overall caloric rate that varies between individuals in the range of 1 to 4 kcal/min [1]. In T2D, GE is frequently disordered, with an even wider inter-individual variation [6], due to frequently delayed [15–17], or accelerated [1, 3], GE in patients with and without chronic complications. By contrast, the rate of GE in a given individual, when assessed with the same test meal, is reasonably reproducible [18–20]. However, it remains to be established whether GE assessed by standardised test ‘meal’ is predictive of the glycaemic response to other physiological meals. For example, a 75 g oral glucose drink is a widely used ‘test meal’ for both the diagnosis of diabetes and also for the assessment of GE. While the glycaemic response to oral glucose is profoundly influenced by GE, the capacity of the latter to predict the glycaemic response to mixed meals is unclear. Understanding this issue is of major importance to the choice of the ‘test meal’ for measurement of GE and to the management of postprandial glycaemia; e.g., current recommendations relating to the glycaemic load and index in T2D are not based on an understanding of the rates of delivery of carbohydrate to the small intestine and are essentially empirical [21]. Similarly, the use of prandial insulin for the management of postprandial hyperglycaemia in type 1 diabetes (T1D) relies mainly on carbohydrate-counting, without consideration of its rate of small intestinal delivery [22, 23].

GE is known to be regulated by a complex set of neurohormonal mechanisms, among which variations in glycaemia have been considered to be a major determinant. Indeed, acute elevations in glycaemia induced by intravenous glucose infusion (i.e., glucose ‘clamping’), even within the physiological postprandial range (~8 mmol/L), delay GE substantially in both health and T1D [24–27]. However, GE in T1D has been reported to be unrelated to day-to-day variations in fasting blood glucose [28], suggesting that spontaneous fluctuations in blood glucose, which occur more slowly than in glucose clamp experiments, are less relevant to the regulation of GE in T1D [29]. In T2D, information about the impact of chronic glycaemic control, assessed by glycated haemoglobin A1c (HbA1c), on GE in T2D remains controversial. In a group of Indian women with newly diagnosed, poorly-controlled T2D who had delayed GE at baseline, a marked improvement in glycaemic control over 2–3 months, achieved by exercise and glipizide, was associated with a profound acceleration of GE [30]. However, in another study, in which T2D patients with poorly-controlled glycaemia and substantial heterogeneity of GE at baseline (either delayed, normal, or accelerated) received exogenous insulin as add-on therapy to their oral glucose-lowering agents over 6 months, GE was unaffected, despite a substantial improvement in HbA1c [31]. Although the current guidelines advocate the need to control fasting blood glucose for the measurement of GE [32], there is a lack of information as to whether spontaneous variations in blood glucose affect GE.

Accordingly, this study examined (i) the relationship of the glycaemic response to both glucose and mixed meals with GE of a 75 g glucose drink, and (ii) the relationship of GE of the glucose drink with markers of antecedent glycaemia, including fasting plasma glucose (FPG), 24-hour glucose profile, serum fructosamine and HbA1c, in newly diagnosed, treatment-naive, Chinese patients with T2D.

## Materials and methods

### Participants

Participants were recruited from the diabetes outpatient clinic of Nanjing First Hospital, Nanjing Medical University, China. All subjects provided written informed consent prior to their participation. Those with significant gastrointestinal symptoms, a history of gastrointestinal disease including known gastroparesis, bariatric surgery, or requiring medication known to affect gastrointestinal function or appetite, were excluded. The protocol was approved by the Human Ethics Committee of Nanjing First Hospital (KY20220124-08) and was prospectively registered on a clinical trials database (NCT05284344).

### Protocol

**Fig. 1:**
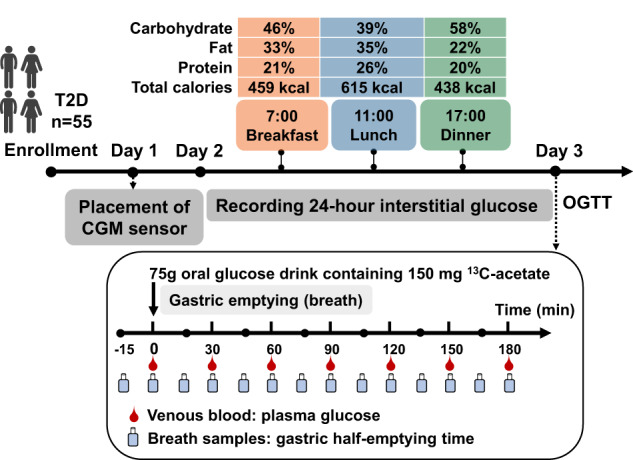
Study protocol. A schematic illustration of the study design depicting the study timeline and study-related activities.

Following enrolment, all participants were admitted to the Clinical Research Unit of the Department of Endocrinology at Nanjing First Hospital for three consecutive days (Fig. 1). On Day 1, a comprehensive medical history was obtained using standardised questionnaires. Microvascular complications of diabetes, including nephropathy, retinopathy and peripheral neuropathy, were assessed by the urinary albumin/creatinine ratio and/or estimated glomerular filtration rate, and clinical examination. A continuous glucose monitoring (CGM) sensor (Medtronic Incorporated, Northridge, Minnesota, USA) was then inserted into the anterior abdominal skin of each participant to provide continuous monitoring of interstitial glucose concentrations. On Day 2, all participants were provided with 3 standardised meals, of breakfast at 0700 h (459 kcal; carbohydrate 46%, fat 33% and protein 21%), lunch at 1100 h (615 kcal; carbohydrate 39%, fat 35% and protein 26%), and dinner at 1700 h (438 kcal; carbohydrate 58%, fat 22% and protein 20%). No other food or liquid, other than water, was allowed throughout Day 2. Capillary blood glucose was measured four times each day for calibration of CGM recordings. On day 3, after an overnight fast, participants consumed a glucose drink on Day 3 (75 g glucose dissolved in water to a final volume of 300 mL) containing 150 mg ^13^C-acetate between *t* = 0 to 5 min. Breath samples were collected immediately before the drink, and every 15 minutes thereafter for 3 hours [3]. Venous blood was sampled immediately before the drink (at *t* = 0), and at *t* = 30, 60, 90, 120, 150, and 180 min, to measure HbA1c and serum fructosamine (*t* = 0) and plasma glucose concentrations. Interventions for T2D were initiated immediately after completion of these evaluations.

### Measurements

Plasma glucose concentrations were measured by the glucose oxidase method using a Hitachi 7600-120 analyser (Hitachi Corp, Tokyo, Japan). HbA1c was measured by a high-performance liquid chromatography assay (Bio-Rad Laboratories Inc., CA, USA), and serum fructosamine using a Glamour 2000 automatic biochemical analyser (MD Inc., CA, USA), yielding a normal range of 200-285µmol/L.

To minimise potential errors arising from insertion and removal of the CGM sensor, Day 2 CGM data (0:00 to 24:00) were used to calculate 24-hour mean interstitial glucose, mean amplitude of glycaemic excursion (MAGE), coefficient of variation (CV), and time in range between 3.9 and 10.0 mmol/L (TIR).

^13^CO_2_ in each breath sample was measured by a HCBT-01 breath test analyser (Headway Bio-Sci & Tech Co., Shenzhen, China) utilising the differences between ^13^CO_2_ and ^12^CO_2_ in the absorption peak of infra-red light. The gastric half-emptying time (T50) was calculated using the Wagner-Nelson method, as described [33]. This method has been shown to be comparable to scintigraphy for measurement of GE of both solid and liquid meals [3, 33–37].

### Statistical analysis

Based on our previous studies, a sample size of 20 subjects would provide at least 80% power to detect significant correlations between the glycaemic response to oral glucose/mixed meals and the rate of GE in patients with T2D [3, 35]. The primary analyses were the relationships of the incremental areas under the curves (iAUCs, calculated by subtracting baseline values from the AUC) for plasma glucose over different time periods after oral glucose and standardised mixed meals with the T50 of the glucose drink. Secondary analyses were the relationships between GE (i.e., T50) and markers of short-, medium- and long-term glycaemic control assessed before the measurement of GE, including (i) FPG and 24-hour mean interstitial glucose, MAGE and TIR, (ii) serum fructosamine, and (iii) HbA1c, respectively. These relationships were assessed using univariate linear regression analysis after confirming that data were normally distributed. All analyses were performed using Prism 9.0 software (GraphPad, La Jolla, CA, USA). Data are presented as means ± SEM; *P* < 0.05 was considered statistically significant.

## Results

**Fig. 2:**
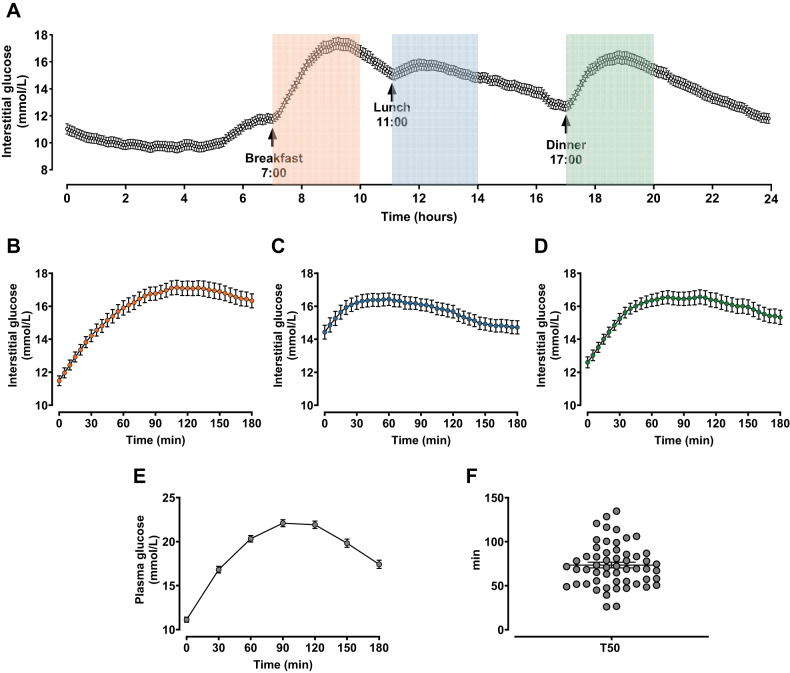
Glycaemic responses to mixed meals and a glucose drink in newly diagnosed, treatment-naive, Chinese patients with type 2 diabetes (T2D). 24-hour interstitial glucose concentrations recorded by continuous glucose monitoring (**A**), and the glycaemic responses to standardised breakfast (**B**), lunch (**C**), and dinner (**D**); plasma glucose concentrations in response to oral glucose (**E**); gastric emptying half-time (T50) of the 75 g oral glucose load (**F**) in newly diagnosed, treatment-naive, Chinese patients with T2D (*n* = 55). Data are mean values ± SEM.

55 newly diagnosed, treatment-naive participants with suboptimal glycaemic control were evaluated, of whom mild diabetic complications were evident in a small subset of participants (*n* = 3 for diabetic nephropathy; *n* = 7 for diabetic neuropathy; *n* = 7 for diabetic retinopathy) (Table 1). All participants tolerated the protocol well. Their interstitial glucose concentrations recorded by a CGM sensor on Day 2 are summarised in Fig. 2A, and consistent with high HbA1c and serum fructosamine levels, 24-hour mean interstitial glucose concentration was 13.4 ± 0.3 mmol/L, while TIR approximated 20%. Interstitial glucose concentrations over 180 min after the standardised breakfast, lunch and dinner, were extracted from the CGM data, and are shown in Fig. 2B–D. Reflecting the persistent hyperglycaemia, the CV of 24-hour interstitial glucose was relatively small. On Day 3, fasting plasma glucose was 11.1 ± 0.3 mmol/L. Following the 75 g glucose drink, plasma glucose increased to a peak of 22.1 ± 0.4 mmol/L at *t* = 90 min, before returning towards baseline (Fig. 2E). The T50 of the glucose drink varied substantially between participants, with a mean of 73.5 ± 3.3 min, and range of 26.0–134.7 min (Fig. 2F).

**Table 1:** Demographic data study outcomes in newly diagnosed, drug-naive Han Chinese patients with type 2 diabetes (T2D).

	T2D
	(*n* = 55)
Demographic data	
Gender (male/female)	38/17
Age (years)	49.5 ± 1.4
BMI (kg/m^2^)	26.6 ± 0.5
HbA1c (%)	9.8 ± 0.2
Serum fructosamine (µmol/L)	427.6 ± 8.1
Diabetic nephropathy	3/55
Diabetic retinopathy	7/55
Diabetic peripheral neuropathy	7/55
CGM data prior to the 75 g oral glucose drink	
24-hour mean interstitial glucose (mmol/L)	13.4 ± 0.3
CV (%)	22.3 ± 1.0
MAGE (mmol/L)	6.3 ± 0.4
TIR (%)	19.7 ± 2.8
75g-OGTT	
Fasting plasma glucose (mmol/L)	11.1 ± 0.3
T50 (min)	73.5 ± 3.3

Data are presented as mean values ± SEM.

*BMI* body mass index; *OGTT* oral glucose tolerance test; 24-hour *CGM* 24-hour continuous glucose monitoring; *CV* coefficient of variation; *MAGE* mean amplitude of glycaemic excursion; *TIR* time in range (percentage of time in the range of 3.9 - 10 mmol/L); *T50* gastric half-emptying time.

### Relationship between glycaemic response and GE after oral glucose

**Fig. 3:**
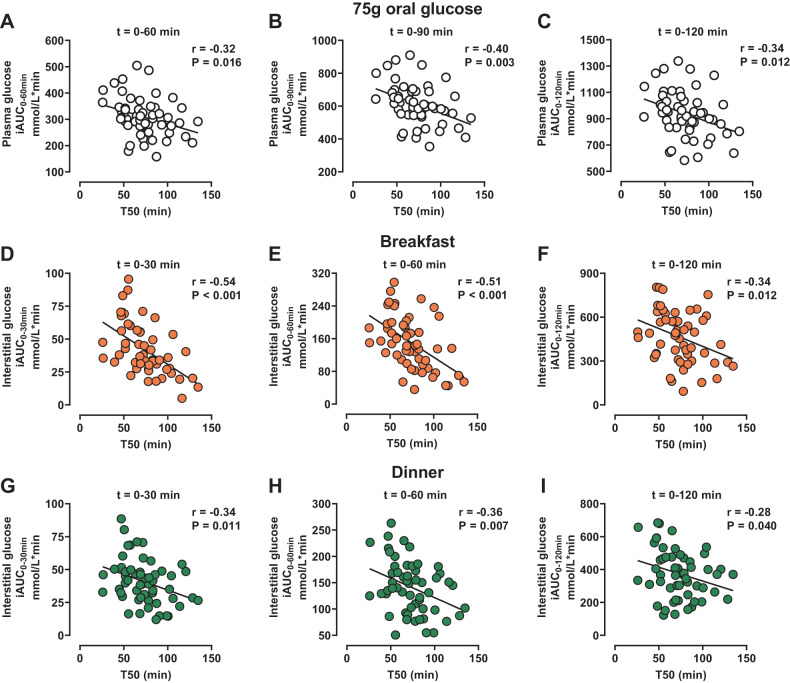
Relationships between the glycaemic responses to oral glucose and mixed meals and gastric half-emptying time of a glucose drink in newly diagnosed, treatment-naive, Chinese patients with type 2 diabetes (T2D). Relationships of (i) the incremental areas under the curves (iAUCs) for plasma glucose between *t* = 0–60 min, *t* = 0–90 min, and *t* = 0–120 min after 75 g oral glucose (**A**–**C**) and (ii) the iAUCs for interstitial glucose, measured by continuous glucose monitoring, between *t* = 0–30 min, *t* = 0–60 min, and *t* = 0–120 min after breakfast (**D**–**F**) and dinner (**G**–**I**), with the gastric half-emptying time (T50) of 75 g oral glucose drink in newly diagnosed, treatment-naive, Chinese patients with T2D (*n* = 55).

After the 75 g oral glucose load, plasma glucose iAUCs between *t* = 0–60 min (*r* = −0.32, *P* = 0.016), *t* = 0–90 min (*r* = −0.40, P = 0.003), and *t* = 0–120 min (*r* = −0.34, *P* = 0.012) were also related inversely to the T50 (Fig. 3A–C).

### Relationship of the glycaemic response to mixed meals with GE of oral glucose

The iAUCs for interstitial glucose after breakfast and dinner were related inversely to the T50 of the glucose drink between *t* = 0–30 min (breakfast: *r* = −0.54, *P* < 0.001; dinner: *r* = −0.34, *P* = 0.011), *t* = 0–60 min (breakfast: *r* = −0.51, *P* < 0.001; dinner: *r* = −0.36, *P* = 0.007), and *t* = 0–120 min (breakfast: *r* = −0.34, *P* = 0.012; dinner: *r* = −0.28, *P* = 0.040) (Fig. 3D–I). However, there was no significant relationship between the iAUCs for interstitial glucose after lunch and the T50 of oral glucose (data not shown). There was also no significant relationship between markers of glycaemic variability over 24-hours (MAGE, CV, and TIR) and the T50 of oral glucose (data not shown).

### Relationship of GE with markers of short-, medium- and long-term glycaemic control

The T50 of oral glucose was not related to FPG (*r* = 0.19, *P* = 0.155) or 24-hour mean interstitial glucose (*r* = −0.02, *P* = 0.896) as assessed by CGM over 24 hours prior to the measurement of GE (Supplementary Fig. 1A, B). The T50 was also unrelated medium-term (i.e., serum fructosamine *r* = −0.05, *P* = 0.704) and longer-term (i.e., HbA1c *r* = 0.06, *P* = 0.686) markers of glycaemic control (Supplementary Fig. 1C, D).

## Discussion

We have demonstrated in newly diagnosed, treatment-naive, Chinese patients with T2D that GE of a 75 g glucose drink is not only predictive of the glycaemic response to the glucose drink but also that of mixed meals, especially breakfast and dinner. In contrast to our expectation, the rate of GE was unrelated to antecedent markers of short-, medium- and longer-term glycaemic control. These observations provide compelling evidence that GE, quantified with a standardised nutrient load, is predictive of the glycaemic response to other carbohydrate-containing nutrient loads, and that the use of a 75 g glucose drink is an appropriate test ‘meal’ for the measurement of GE in this context. These insights are of relevance to recommendations relating to the glycaemic index and load of carbohydrate in T2D and, presumably, impaired glucose tolerance which, in general, lack a mechanistic basis [21], and support the rationale for the use of dietary [7–9] and pharmacological [10] strategies, which slow GE, to reduce postprandial glycaemic excursions.

The participants had a mean age of ~50 years and HbA1c of 9.8%, with only a small proportion demonstrating evidence of microvascular complications. These features are typical of the clinical profile of newly diagnosed T2D patients in China [38]. We recognised that in this group of T2D patients, fasting blood glucose, rather than postprandial glycaemia, would be the dominant determinant of overall glycaemic control [39]. Despite this, we observed that the iAUCs for plasma glucose between *t* = 0–60 min, 0–90 min and 0–120 min after 75 g glucose drink were related inversely to the T50 (i.e., related directly to the rate of GE). This finding is consistent with the observations made in T2D patients with relatively well control glycaemia [1], although the correlation coefficients were weaker in our current cohort. Our previous work has shown that the initial increments in plasma glucose following an oral glucose load are reflective of glucose appearance, which is regulated primarily by GE [40, 41]. The lack of significant correlations between plasma glucose iAUC and the T50 beyond 2 hours may have reflected the introduction of other mechanisms responsible for glucose disposal (e.g., the secretion and action of insulin) in the later phase [2, 42]. Nevertheless, our observations have demonstrated a sustained impact of GE on the glycaemic response to the oral glucose load in newly diagnosed, treatment-naive Chinese patients with T2D.

Importantly, GE of oral glucose was predictive of the blood glucose response to more physiological mixed meals. As with the observations made during the oral glucose tolerance test (OGTT), the iAUCs for interstitial glucose within 2 hours after breakfast and dinner were related inversely to the T50 of oral glucose. While it was already appreciated that GE in a given individual is relative stable [18–20], the current study provides the first demonstration that GE determined by a 75 g oral glucose load was effective to predict the glycaemic response to breakfast and dinner in T2D. The lack of a significant correlation between the glycaemic response to lunch and the T50 of oral glucose is likely to be attributable to incomplete emptying of breakfast from the stomach by the time lunch was ingested, reflected by substantially higher glucose concentrations prior to lunch than those immediately before breakfast and dinner. Accordingly, concurrent measurement of GE during OGTT is relevant to understanding the mechanisms responsible for postprandial hyperglycaemia and informing therapeutic strategies to optimise postprandial glycaemic control in T2D.

It is intuitive that GE might also have predicted glycaemic variability, but neither MAGE nor CV derived from the 24-hour CGM correlated with the T50 of oral glucose. However, this should not be surprising, since the patients in the current cohort had markedly elevated HbA1c; in such patients, fasting hyperglycaemia predominates over postprandial blood glucose in determining overall glycaemic control [39]. Consistent with this notion, the CV for 24-hour interstitial glucose was only ~22% (i.e., lower than the therapeutic target of 36% recommended by the American Diabetes Association guideline [43]). It would be of interest to examine whether correlations between glycaemic variability and GE become more apparent in T2D patients with better glycaemic control, where postprandial hyperglycaemia is the major determinant of HbA1c. Given the relatively small sample size, a type II error could not be ruled out. Nevertheless, this study showed consistent relationships of glycaemic responses to oral glucose and mixed meals (breakfast and dinner) with GE in Chinese T2D patients.

While GE is recognised to be a key determinant of postprandial glycaemia in both health and T2D [44, 45], it remains uncertain as to whether everyday variations in glycaemia, in turn, influence GE. In the current cohort, GE was found to be unrelated to the preceding FPG, 24-hour mean interstitial glucose assessed by CGM, serum fructosamine, or HbA1c. These observations do not support a significant impact of spontaneous glycaemia on GE. Indeed, in T2D patients with few complications, GE is often more rapid than in healthy subjects, regardless of their HbA1c levels [3]. Similarly, no significant correlation was observed between GE and spontaneous day-to-day variations in FPG in patients with T1D [28]. However, abrupt increments in glycaemia induced by intravenous glucose infusion were shown to induce substantial slowing of GE in both health and T1D [24–27]. The discrepancy is likely to be related to the rate of change in plasma glucose; prompt changes in plasma glucose have the potential to disrupt the balance between the sympathetic and parasympathetic autonomic nervous system, leading to acute changes in GE [46], whereas fluctuations of plasma glucose occurring more slowly may have allowed counterregulatory or adaptive responses, leading to the absence of a relationship between GE and spontaneous glycaemic variations. Consistent with this, several interventional studies failed to show a link between GE and preceding glycaemic control [30, 31, 47]. Taken together, we believe that slow changes in glycaemia per se do not affect GE substantially in T1D and T2D. Further validation of this concept is important, since variations in the rate of GE have major implications for the management of both diabetic gastroparesis and postprandial hyperglycaemia.

Several limitations in the current study should be noted. First, the sample size may be perceived modest, but was sufficient to demonstrate consistent relationships between the glycaemic responses to both oral glucose and mixed meals and GE of oral glucose. Second, in order to minimise potential confounders, only newly diagnosed, treatment-naive patients with T2D were studied. Accordingly, it remains unclear whether GE in this cohort of patients differs from appropriately matched healthy controls, and generalisation of our findings to the broader T2D community should be undertaken with caution. Future studies involving patients with different degrees of glycaemic control, and receiving different glucose-lowering therapies, are warranted. Third, the present study was observational only. Interventional studies are warranted to determine whether modulation of glycaemia in different settings affects GE in T2D. Finally, we could not obtain reliable information on dietary habits in individual patients, which may also account for the wide variation in GE between individuals [48, 49].

## Conclusion

In summary, our study has demonstrated that in newly diagnosed, treatment-naive, Chinese patients with T2D, GE of a 75 g glucose drink predicts the glycaemic response to other more physiological meals, particularly breakfast and dinner, and is not influenced by the spontaneous variations in blood glucose in either the short, medium or longer term. These findings support concurrent measurement of GE during a 75 g OGTT, which can be achieved relatively easily with a stable isotope breath test, to inform the management of postprandial glycaemia in Chinese patients with T2D. Moreover, measurement of GE may not be limited by spontaneously developed hyperglycaemia.

### Supplementary information

Supplementary Figure

## Data Availability

The datasets generated during and/or analysed during the current study are not publicly available but are available from the corresponding author on reasonable request.
