# Supplementary material for: Gastric emptying of a glucose drink is predictive of the glycaemic response to oral glucose and mixed meals, but unrelated to antecedent glycaemic control, in type 2 diabetes
Source: Nutr Diabetes. 2024 Apr 8;14:13. doi: 10.1038/s41387-024-00264-8 (PMC11001856; doi:10.1038/s41387-024-00264-8)

**Supplementary Figure 1:** **Relationships between gastric emptying and the markers of short-, medium- and long-term glycaemic control in newly-diagnosed, treatment-naïve, Chinese patients with type 2 diabetes (T2D).** Relationships of the gastric half-emptying time (T50) with fasting plasma glucose (A), 24-hour mean interstitial glucose (B), fructosamine (C), and HbA1c (D) in newly-diagnosed, treatment-naïve, Chinese patients with T2D (n = 55).


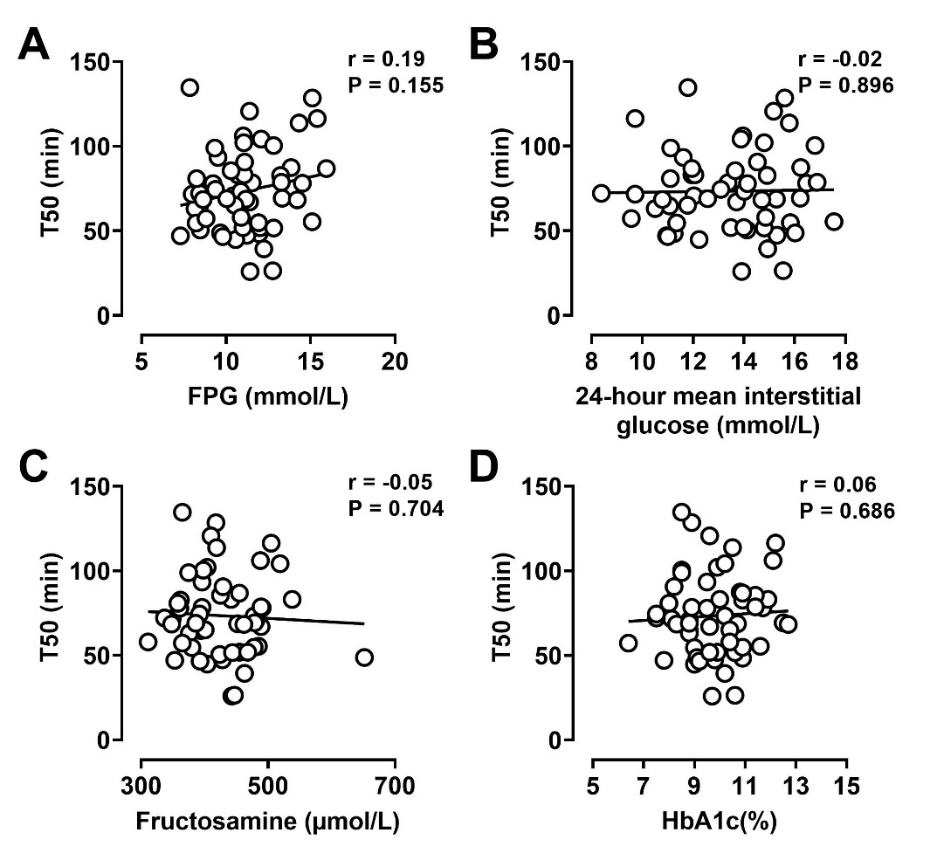

Supplement: Supplementary file 1 — Supplementary Figure [file 41387_2024_264_MOESM1_ESM.docx]
